# Dynamics, function, and regulation of mammary stem cells in development and disease

**DOI:** 10.1038/s12276-026-01780-6

**Published:** 2026-08-03

**Authors:** Eunmi Lee, Yujie Tao, Alice J. Wang, Yibin Kang

**Affiliations:** 1 Department of Molecular Biology, Princeton University, Princeton, NJ USA; 2 Ludwig Institute for Cancer Research Princeton Branch, Princeton, NJ USA; 3 Department of Microbiology, Yeungnam University College of Medicine, Daegu, Republic of Korea; 4 Cancer Metabolism and Growth Program, Rutgers Cancer Institute, New Brunswick, NJ USA

**Keywords:** Breast cancer, Mammary stem cells

## Abstract

Understanding the heterogeneity of mammary epithelial cells and their contributions to postnatal development and regeneration is of great importance. Recent advances have defined the intrinsic complexity of the mammary epithelium, its stromal interactions, and the ontogeny of adult stem and progenitor cells. These insights have not only refined our understanding of normal mammary gland biology but also highlighted how specific epithelial subsets serve as cells of origin and targets of transformation in breast cancer. The close parallels between mammary stem cells and tumor-initiating cells, particularly their shared plasticity and self-renewal, highlight stemness as a central node linking developmental programs to malignant transformation. Both populations are shaped by microenvironmental cues and specialized niche components that regulate their maintenance, fate, and susceptibility to oncogenic reprogramming. Here, we review recent advances in mammary epithelial biology, emphasizing the key signaling pathways and stromal interactions that govern mammary stem cell behavior during development and tumorigenesis. We further discuss how developmental programs and microenvironmental cues that sustain mammary morphogenesis are subverted during cancer progression, with particular attention to pregnancy-associated transient susceptibility and aging-associated cancer risk. Overall, this Review offers a framework for understanding how normal mammary epithelial programs are redirected during malignant progression.

## Microenvironment shapes mammary stem cells and tumor growth

This Review delves into the complex biology of mammary gland development and its implications for breast cancer. It highlights the dynamic nature of mammary epithelial cells, particularly mammary gland stem cells, which possess self-renewal and multipotency capabilities. Recent advances in single-cell transcriptomics and lineage tracing have revealed unexpected diversity and plasticity within these cell populations. The authors discuss how mammary gland stem cells are regulated by intrinsic factors such as TP53 and signaling pathways such as Wnt, and how these elements are often hijacked during tumorigenesis. This Review also explores the impact of physiological changes, such as pregnancy and aging, on the mammary microenvironment, creating windows of increased cancer susceptibility. These insights provide a framework for understanding breast cancer heterogeneity and highlight potential therapeutic targets.

This summary was initially drafted using artificial intelligence, then revised and fact-checked by the author.

## Introduction

The mammary gland represents a unique model of postnatal organ development, whose primary physiological role is milk production for offspring nourishment^1–3^. Structurally, it is composed of epithelial, adipose, and various stromal cells, including fibroblasts, endothelial cells, and immune cells that work in concert to achieve this function^4^. The mammary gland undergoes much of its morphogenesis after birth. In the mouse, five pairs of rudimentary epithelial placodes arise from the ectoderm around embryonic day 10.5 and expand until embryonic day 18, after which further growth is largely restricted until puberty^1^. At puberty, extensive ductal elongation and branching morphogenesis occur, forming a mature ductal tree that continues to remodel throughout adulthood in response to hormonal and physiological cues. Ductal outgrowth is driven by club-shaped, highly proliferative terminal end buds (TEBs) that invade the adipose-rich mammary fat pad. Each TEB consists of an outer layer of cap cells, which give rise to basal myoepithelial cells, and an inner layer of body cells, which differentiate into luminal cells. As ductal elongation completes and the growing tips reach the boundary of the mammary fat pad, TEBs regress, leaving a mature ductal network composed of primary ducts, secondary branches, and tertiary side branches that extend laterally to fill the gland^5^. Cyclical remodeling of the mammary epithelium continues throughout life. The mature gland remains remarkably dynamics, undergoing periodic structural and functional changes in response to hormonal fluctuations. During estrus cycle, subtle rounds of ductal budding and regression occur, maintaining epithelial turnover and plasticity. With pregnancy, the gland undergoes extensive lobuloalveolar expansion as luminal progenitors (LPs) proliferate and differentiate into milk-secreting alveolar cells, while basal–myoepithelial cells acquire contractile specialization to facilitate milk ejection. After lactation and breastfeeding, the withdrawal of hormonal and mechanical stimuli triggers a highly coordinated involution process characterized by epithelial apoptosis, tissue remodeling, and immune cell infiltration, ultimately restoring the gland to a near-virgin architecture that is poised for subsequent cycles of reproduction^1,3^.

Although these developmental dynamics are best characterized in mice, the human mammary gland exhibits a similar architecture and remodeling pattern, underscoring the evolutionary conservation of mammary morphogenesis. The functional unit of the human breast, the terminal ductal lobular unit (TDLU), serves as the counterpart to the murine TEB, alveolar structures. Each TDLU comprises a network of small ductules and acinar structures embedded within a specialized stroma. In the virgin gland, relatively undifferentiated Lob1-type TDLUs predominate and are thought to correspond functionally to the proliferative TEBs in mice. With sexual maturation and reproductive cycles, these units develop into Lob2-type structures characterized by increased ductular complexity. During pregnancy, further branching and alveolar differentiation give rise to Lob3-type and Lob4-type TDLUs, which form secretory acini specialized for milk production. After lactation, extensive regression and remodeling return the gland predominantly to a Lob2 state, whereas postmenopausal atrophy leads to a re-emergence of Lob1-type units, marking a return to a quiescent architecture^6,7^.

These cyclical, hormonally orchestrated remodeling events point to the existence of long-lived mammary stem cells (MaSCs) endowed with self-renewal and multilineage differentiation potential^8^. To sustain repeated cycles of pregnancy, lactation, and involution, the putative MaSCs and their progenies must continually regenerate the ductal and alveolar epithelium with remarkable regenerative capacity. This dynamic regenerative system is proposed to be governed by a finely tuned hierarchy in which stem and progenitor cells respond to hormonal cues and paracrine signals integrated within the tissue microenvironment^9^. Crosstalk between epithelial cells and their surrounding stromal, immune, and vascular components not only maintains tissue homeostasis but also coordinates morphogenesis and regeneration throughout life. However, the same developmental plasticity that underlies the regenerative potential of the mammary gland may also confer vulnerability to oncogenic reprogramming. Accumulating evidence suggests that dysregulation of mammary stem or progenitor cell function, or disruption of their communication with the microenvironment, can initiate malignant transformation^10^. Perturbations in developmental hierarchies and epithelial-stromal signaling networks are now recognized as key drivers of tumor initiation, subtype specification, and progression in breast cancer, linking physiological remodeling with pathological transformation. Recent advances in lineage tracing, single-cell transcriptomics, and spatial profiling have begun to unravel the complexity of the mammary epithelial hierarchy, redefining the identity, lineage potential, and niche dependence of mammary stem and progenitor cells^11^. These studies provide critical insights into how developmental programs are hijacked or reactivated during tumorigenesis, linking normal stem cell biology to breast cancer initiation and progression.

In an earlier review, we outlined the discovery of MaSCs and their proposed contribution to breast cancer initiation^3^. Here, we revisit this topic to highlight recent advances in mammary epithelial cell (MEC) biology, emphasizing the key signaling pathways and stromal interactions that regulate MaSC behavior during normal development and tumorigenesis. We further discuss how developmental programs and microenvironmental cues that sustain mammary morphogenesis are hijacked during cancer progression, with particular attention to the transient increase in breast cancer susceptibility observed in high-risk individuals during and after pregnancy as well as the link between cancer and aging. By integrating insights from developmental biology, stem cell research, and cancer biology, this Review provides a conceptual framework for understanding how “milk-making” cells can be reprogrammed toward malignancy.

## Identification of mammary gland stem cells and their progeny

Given its unique postnatal morphogenesis, dynamic developmental plasticity, and importance to women’s health and nutrition, the characterization of MECs and their functional heterogeneity have long been a subject of intense investigation. Particular interest has centered on mammary gland stem cells (MaSCs) because of their specialized properties of self-renewal and multipotency, which enable them to generate the diverse lineages, including luminal secretory, luminal hormone-sensing, and basal/myoepithelial lineages that compose the mammary epithelium^12,13^. Despite decades of research and the establishment of classical stem cell assays, our understanding of MaSC identifying lineage relationships has undergone a major revision in recent years. Advances in single-cell transcriptomic profiling and in vivo lineage-tracing analyses have uncovered unexpected diversity among MEC populations, revealing context-dependent fate plasticity and dynamic cellular hierarchies. The evolving taxonomy and nomenclature of MECs emerging from these studies exemplify the remarkable phenotypic and functional heterogeneity within the mammary epithelium^11^.

Historically, the identification of stem cell population relied heavily on transplantation-based assays, most notably the cleared fat pad reconstitution model developed by DeOme et al. in the 1950s^14^. In this system, small fragments or clusters of mammary epithelia were transplanted into epithelial-free fat pads of recipient mice, where they regenerated a complete and functional ductal tree, thereby demonstrating stem cell potential. Subsequent limiting-dilution and lineage-specific marker analyses refined the isolation of MaSC-enriched populations^15^. The advent of fluorescence-activated cell sorting allowed researchers to fractionate MECs based on surface marker expression, leading to the identification of basal-enriched MaSC populations characterized by a Lineage⁻CD24⁺CD29^high^ or Lineage⁻CD24⁺CD49f^high^ phenotypes, which are separated from Lineage⁻CD24⁺CD29^low^ or Lineage⁻CD24⁺CD49f^low^ luminal cells. Transplantation of single cells from these subsets into cleared fat pads was sufficient to regenerate an entire epithelial tree, definitively demonstrating their self-renewal and multilineage differentiation potential^2,12^. These MaSCs reside predominantly within the basal compartment, adjacent to the basement membrane, and gene expression analyses confirmed that MaSC-enriched basal populations express canonical myoepithelial markers such as keratins 5 and 14 (K5 and K14) and smooth muscle actin while maintaining transcriptional programs associated with stemness and tissue remodeling^13,16^. Efforts to prospectively identify and isolate MaSCs using transplantation-based and flow cytometry-based assays remain the gold standards in the field. More recently, 3D organoid culture systems have emerged as powerful complementary tools to study MaSC biology ex vivo^17^. In these systems, basal cells generate complex bilayered organoids containing both luminal and myoepithelial layers, whereas luminal cells give rise to lineage-restricted acini, faithfully recapitulating in vivo tissue architecture^18^. However, such assays have also raised questions about their physiological relevance, as regeneration under artificial conditions may not fully capture stem cell behavior within the native tissue niche. Moreover, variables such as the method of MEC isolation, FACS strategy, extracellular matrix (ECM) supplementation, and culture media formulation can influence observable plasticity and clonogenic potential during normal differentiation^11^.

**Fig. 1:**
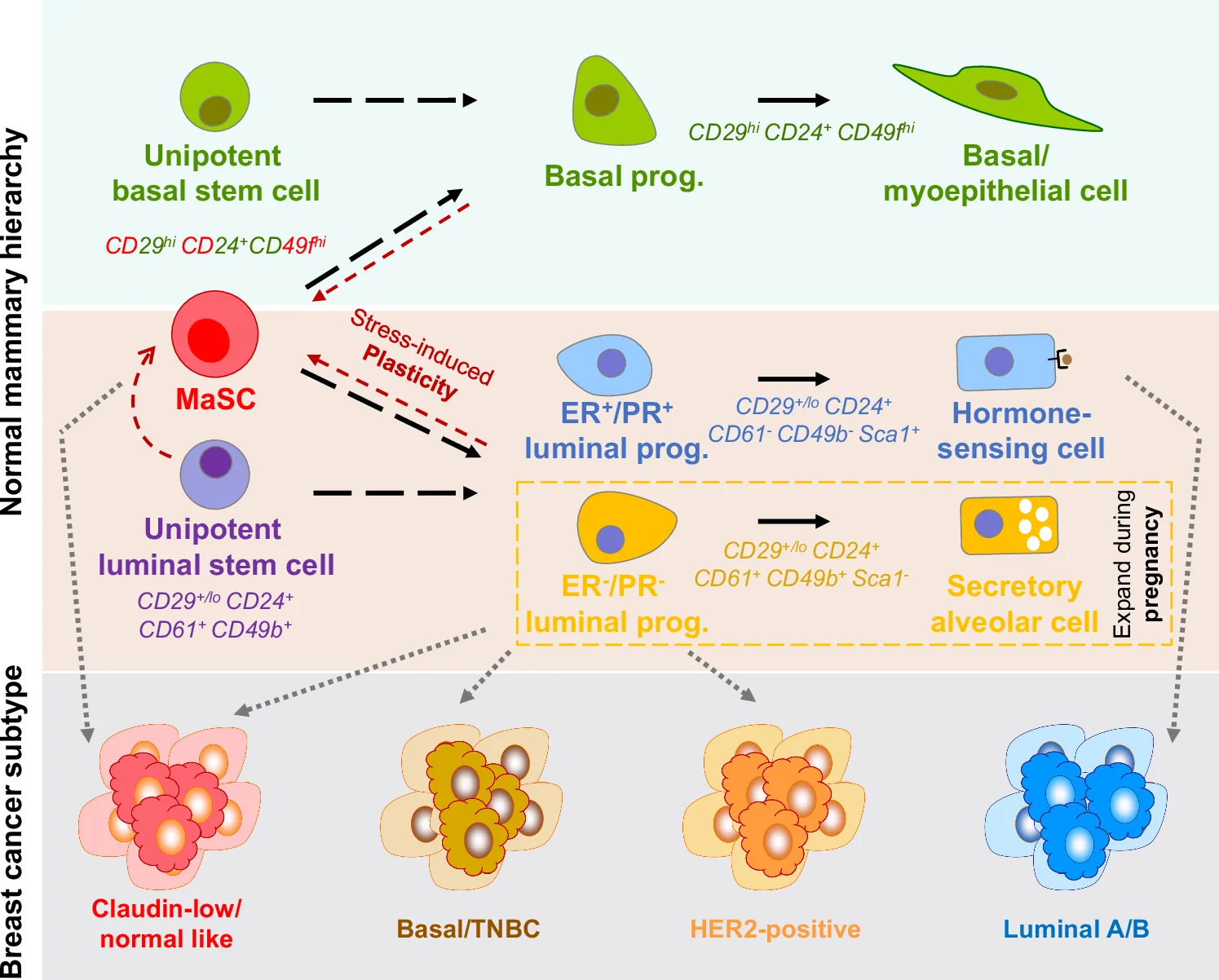
Mammary epithelial hierarchy, lineage relationships, and plasticity in normal development and breast cancer. The mammary epithelium is organized into basal and luminal lineages maintained by lineage-restricted basal and luminal stem/progenitor cells, as well as a multipotent mammary stem cell (MaSC) population. Basal/myoepithelial cells arise from basal progenitors (prog.), whereas hormone-sensing cells differentiate from ER^+^/PR^+^ luminal progenitors (LPs) and secretory alveolar cells derived from ER^−^/PR^−^ LPs. Representative cell surface markers are indicated. Basal lineage cells, including MaSCs, are characterized by CD29^hi^, CD49f^hi^, and CD24^+^. LP cells are defined by CD61 expression with CD29^+/lo^, CD24^+^. ER^−^/PR^−^ LPs are additionally marked by CD49b^+^, Sca1^−^, whereas ER^+^/PR^+^ LPs are Sca1^+^ and typically lack CD61 and CD49b expression. Differentiated secretory alveolar cells downregulate CD49b and CD61 expression. These markers are widely used to distinguish functionally distinct mammary epithelial subpopulations in mouse models. Physiological expansion of ER^−^/PR^−^ LPs and secretory alveolar cells occurs during pregnancy. In addition, dashed red arrows denote stress-induced plasticity, including transitions between basal and luminal states and reacquisition of stem-like features. Major breast cancer subtypes are depicted below, reflecting their closest putative cells of origin: claudin-low/normal-like (MaSC-associated), basal/triple-negative breast cancer (TNBC), HER2-positive (LP-associated), and luminal A/B (hormone-sensing luminal lineages). Dashed arrows indicate potential lineage plasticity. ER, estrogen receptor; PR, progesterone receptor.

The advent of in vivo lineage tracing using Cre/loxP-based reporter systems revolutionized the field by enabling the tracking of specific cell populations during normal development^19^. These studies revealed that mammary epithelial hierarchy is far more dynamic than previously appreciated, challenging the notion of a single, long-lived multipotent MaSC and instead suggesting the existence of lineage-restricted stem and progenitor populations with context-dependent plasticity (Fig. 1). Although different gene promoters have been utilized for lineage-tracing studies, basal cell mapping has yielded discrepant results. Lineage tracing driven by generic basal markers such as K5, K14, and smooth muscle actin, as well as Axin2 and Lgr5 promoters, demonstrated the presence of long-lived, basal-restricted unipotent progenitors that drive postnatal ductal morphogenesis^20^. These findings are supported by independent studies showing that unipotent LPs maintain the luminal lineage, suggesting that the two epithelial compartments do not normally transdifferentiate under homeostatic conditions^21^. By contrast, other lineage-tracing analyses using the same basal markers (K5 and Lgr5) or more restricted stem-cell-associated promoters such as Procr and Dll1 have provided evidence for a population of long-lived multipotent stem cells in adult mammary glands capable of generating both basal and luminal lineages^22–24^. To mitigate the limitations of promoter specificity, stochastic lineage-tracing models have been developed to track cell fate in a promoter-independent manner^25^. Although these models primarily detect unipotent cells under physiological conditions, they rely on DNA replication for labeling and thus may overlook quiescent stem cells, a hallmark of many tissue-resident stem cell populations^2,26^. Recent studies have begun to reconcile these discrepancies by revealing previously unrecognized spatial and transcriptional heterogeneity within the basal compartment. A recent single-cell and spatial transcriptomic analysis identified three spatially distinct basal-MaSC subpopulations based on CD34 and CD200 expressions. CD34^−^CD200^+^ basal cells, enriched near the nipple region, exhibited the highest self-renewal and repopulating capacity, whereas CD34^+^CD200^−^ cells, localized primarily in TEBs, displayed reduced potency^27^. These findings highlight that MaSC potential varies by anatomical location and local signaling context, potentially explaining the divergent conclusions of lineage-tracing studies.

A luminal hierarchy model describes functionally and morphologically distinct cell types defined by specific surface markers and lineage potential. Early flow cytometry and colony-forming assays identified CD61⁺ (integrin β3) cells within the Lineage⁻CD24⁺CD29^low^ population as LPs, whereas CD61⁻ cells represented more differentiated, hormone receptor-expressing luminal cells (estrogen receptor (ER)). Subsequent refinement using Sca1 and CD49b expression further delineated two main LP subsets: ER⁺ (Sca1⁺CD49b⁺) and ER⁻ (Sca1⁻CD49b⁺) progenitors, whereas Sca1⁺CD49b⁻ cells corresponded to non-clonogenic, hormone-sensing mature luminal cells. CD61⁺ LPs are largely ER⁻, enriched for milk-synthesis and secretory gene programs, functioning as major alveolar precursors, whereas mature luminal cells display hormone-responsive gene signatures^28^. Lineage-tracing studies have provided direct evidence that unipotent progenitors sustain the luminal lineages under physiological conditions. Markers such as Elf5, Blimp1, Notch1/3, Wap, and Sox9 define long-lived ER⁻ progenitors that expand during pregnancy and give rise to mature secretory alveolar cells, whereas ER⁺ progenitors marked by Prom1/CD133 or Esr1 self-renew independently to maintain the ductal ER⁺ lineage^20,21,29^. These observations indicate that ER⁺ and ER⁻ luminal branches are maintained by distinct progenitor pools with minimal interconversion during postnatal homeostasis (Fig. 1). Interestingly, human breast tissues contain a higher proportion of ER⁺ luminal-restricted progenitors (~25%) compared with mouse glands (~5%), and human LPs frequently co-express basal markers such as K5 and K14, reflecting hybrid basal–luminal phenotypes within TDLUs^2,30^. In an organoid study, EpCAM^+^CD49f^+^ LPs from human breast were able to generate both mature luminal and basal cells, and immunofluorescence staining confirmed co-expression of lineage specific markers in organoids derived from single LPs^18^. These findings underscore the evolutionary diversification of luminal programs between mouse and human mammary glands.

Collectively, studies integrating flow cytometry, transplantation, lineage tracing, and transcriptomic profiling demonstrate that the mammary epithelial hierarchy is heterogeneous, dynamic, and context-dependent rather than fixed. The current consensus is that unipotent progenitors primarily drive postnatal morphogenesis and adult tissue maintenance, whereas multipotent MaSCs support long-term ductal integrity and regeneration. Under regenerative, hormonal, or pathological stress, however, both basal and luminal cells may exhibit facultative stem cell behavior, reacquiring stem-like potential, similar to the adaptive regeneration observed in injury-prone tissues such as the intestine or skin^2,9,31^. Ultimately, this dynamic plasticity, shaped by spatial, developmental, and microenvironmental cues, is indeed a defining feature of mammary gland biology and forms a critical conceptual bridge between normal regeneration and tumor initiation.

## Linking mammary epithelial hierarchy to the cellular origins of breast cancer

As one of the leading causes of cancer mortality in women, breast cancer exhibits extensive heterogeneity at pathological, genetic, and microenvironmental levels, resulting in diverse clinical manifestations and variable therapeutic responses^32^. Breast cancer encompasses a broad spectrum of histopathological entities distinguished by their cellular morphology, architectural patterns, and molecular characteristics. Preinvasive lesions such as ductal carcinoma in situ and lobular carcinoma in situ represent key precursors in tumor progression. The majority of invasive tumors are classified as invasive ductal carcinoma of no special type, accounting for ~70–80% of cases, whereas invasive lobular carcinoma constitutes about 10–15% and is characterized by E-cadherin-deficient discohesive tumor cells with reduced intercellular adhesion and appearing as individual cells or loose clusters rather than a tightly bound tissue structure^33^.

Integration of histopathological and molecular classifications provides greater prognostic precision and guides personalized therapeutic strategies. Importantly, distinct molecular and intrinsic subtypes of breast cancer mirror the biological diversity of the mammary epithelium and its oncogenic trajectories (Fig. 1). Broadly, breast cancers are categorized as luminal A, luminal B, HER2-enriched, basal-like (triple-negative), and claudin-low subtypes^7^. Luminal A and luminal B tumors, typically ER/PR-positive, originate from luminal epithelial lineages, differing mainly in proliferative index and responsiveness to endocrine therapy. Luminal A tumors are generally low grade, strongly ER+PR+, express luminal genes (ESR1, GATA3, XBP1, and FOXA1), and are associated with favorable outcomes, whereas luminal B tumors exhibit reduced luminal gene expression, higher proliferation activity, and poorer prognosis^32^. The HER2-enriched subtype, defined by ERBB2 amplification and PI3K-AKT pathway activation, is clinically aggressive yet responsive to anti-HER2 targeted therapies^7^. Although its precise cell of origin remains incompletely defined, this subtype is heterogeneous in hormone receptor expression, with most cases being ER-negative but a subset retaining ER or PR positivity. Most HER2-enriched tumors share a core HER2-regulated transcriptional program, suggesting a common oncogenic signaling pathway rather than a single lineage origin^34^. Nonetheless, evidence from MMTV-neu mouse models indicates that HER2 overexpression in luminal epithelial or LP cells efficiently induces tumors resembling human HER2-enriched cancers^35^. Basal-like or triple-negative breast cancers lack ER, PR, and HER2 expression, often express basal cytokeratins (CK5/6 and CK14), and are thought to arise from LP cells that undergo lineage reprogramming and dedifferentiation following oncogenic insults such as BRCA1 or TP53 loss^36^. Similarly, the claudin-low subtype, initially proposed to originate from MaSCs, is now thought to possibly arise from LPs that acquire mesenchymal and stem cell-like properties through oncogenic signaling, such as RAS activation^37^. In sum, the epithelial hierarchy provides a structured yet flexible landscape in which oncogenic drivers can generate diverse breast cancer subtypes. Importantly, these distinct oncogenic events often influence stem-cell-associated programs, many of which are shared by MaSCs and tumor-initiating cells (TICs). We next review the intrinsic factors, signaling pathways, and microenvironmental regulators that govern these closely related cell populations.

## Shared and distinct molecular regulators of MECs and tumor-initiating cells

The concept of TIC, also referred to as the cancer stem cell, in breast cancer emerged in 2003, when Al-Hajj et al.^38^ demonstrated that a small subset of Lineage^−^CD44^⁺^CD24^−/low^ human breast cancer cells could efficiently initiate tumors in NOD/SCID mice. Since then, it has been widely proposed that MaSCs and TICs represent two ends of the same biological spectrum: MaSCs sustain normal mammary gland development, remodeling, and homeostasis^12^, whereas TICs hijack these stem cell programs to drive tumor initiation, progression, and metastasis. Both cell types exhibit self-renewal, multipotency, and phenotypic plasticity, governed by shared molecular regulators that control stem cell dynamics^10,39^.

## Intrinsic factors

**Fig. 2:**
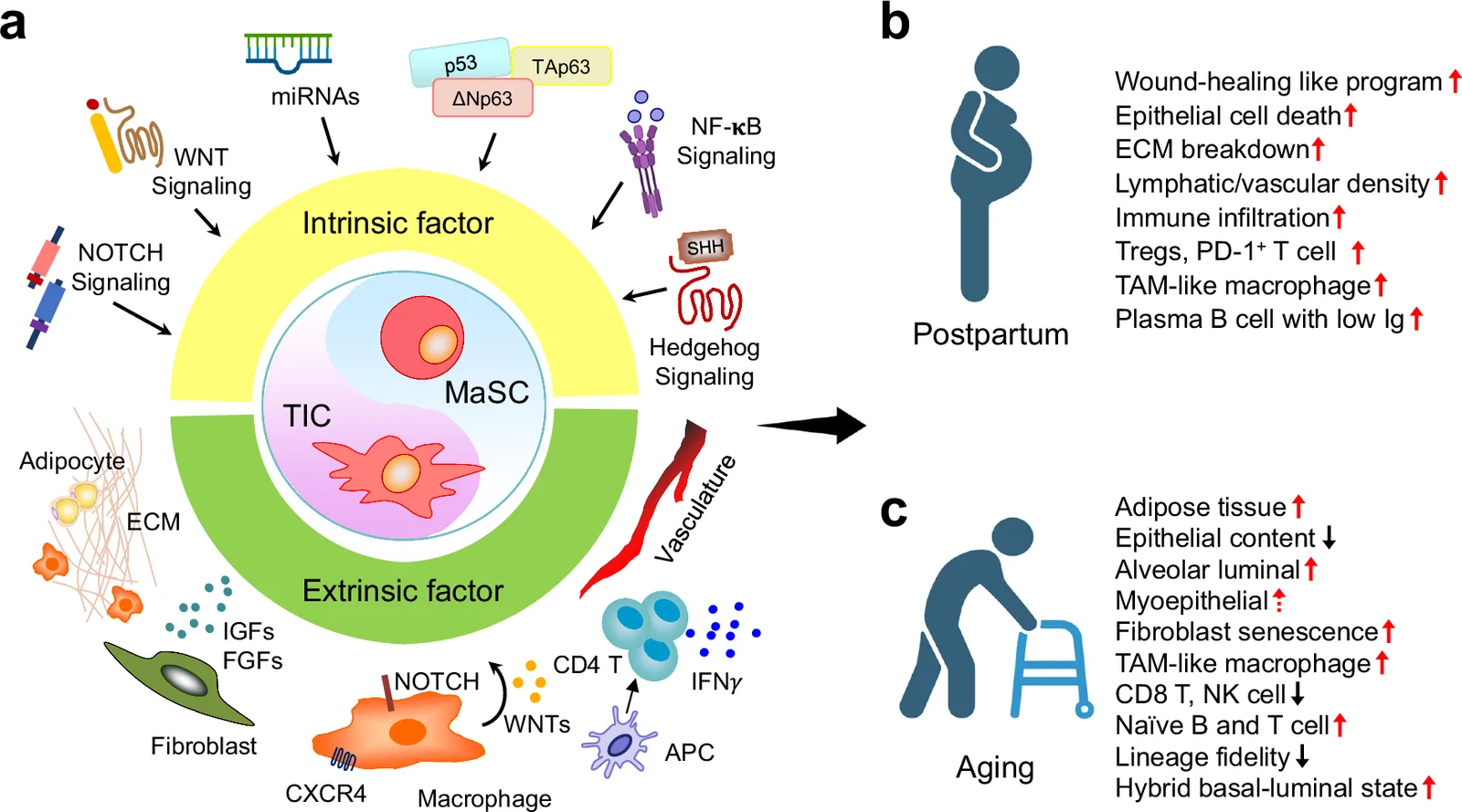
Intrinsic and extrinsic regulators of MaSC and TIC function, and physiological states that remodel the mammary gland microenvironment. **a** Key intrinsic pathways, including Wnt, Notch, Hedgehog, nuclear factor (NF)-kB signaling, p53/p63 isoforms, and miRNAs, together with extrinsic cues from fibroblasts, adipocytes, vasculature, and immune cells collectively regulate mammary stem cell (MaSC) and tumor-initiating cell (TIC) behavior. **b** Postpartum mammary gland remodeling recapitulates key stages of involution, generating a transient wound-healing-like niche characterized by extensive extracellular matrix (ECM) remodeling, lymphangiogenesis, and diverse inflammatory signals. **c** Aging induces adipose expansion, epithelial attrition, fibroblast senescence, immune remodeling, reduced lineage fidelity, and increased hybrid basal–luminal states. The illustration was created in part with BioRender.com. APC, antigen-presenting cell; FGF, fibroblast growth factor; IFN, interferon; IGH, insulin-like growth factor; miRNA, microRNA; SHH, sonic hedgehog; TAM, tumor-associated macrophage; T_reg_, regulatory T.

Intrinsic regulators of MECs comprise transcription factors, epigenetic modulators, non-coding RNAs, and core signaling pathways that collectively govern the behavior, plasticity, and fate of both MaSCs and TICs (Fig. 2a). These factors are discussed within a factor-based framework to highlight shared molecular programs as well as context-dependent functions, while recognizing that they operate within integrated regulatory networks that coordinate stem cell behavior.

## TP53

TP53 is a central tumor suppressor that safeguards genome integrity by coordinating DNA damage repair, cell-cycle arrest, senescence, and apoptosis, metabolism, and chromatin remodeling. In the normal breast, TP53 restricts MaSC expansion and self-renewal, therefore preserving epithelial homeostasis^40,41^. Serial transplantation of TP53-null mammary tissue generates immortal, hormone receptor-positive preneoplastic outgrowths that progress from hyperplasia to invasive carcinoma. Mechanistically, TP53 restricts TIC formation by suppressing stemness-associated transcriptional programs, including Myc, Sox2, and Oct4 (refs. ^42,43^). In human breast cancer, TP53 mutations are highly prevalent, particularly in basal-like and triple-negative subtypes. Mutant TP53 not only loses tumor-suppressive function but also often acquires gain-of-function properties that enhance TIC self-renewal and drive aggressive tumor initiation and progression^44^. Together, these findings highlight TP53 as a key molecular barrier to MaSC transformation and underscore its critical role in restraining TIC emergence and breast cancer evolution.

## TA-p63 and ΔN-p63

p63 produces two principal isoforms: TA-p63, which contains an N-terminal transactivation domain, and ΔN-p63, which lacks this domain. These isoforms have distinct yet complementary roles in regulating mammary epithelial stemness^45^. ΔN-p63 is predominantly expressed in basal epithelial and progenitor cells and is essential for MaSC maintenance and self-renewal^46^, whereas TA-p63 is enriched in luminal cells and promotes differentiation while limiting stem cell expansion^47^. Loss of ΔN-p63 recapitulates the ductal defects observed in *p63*-null mice, whereas TA-p63 deletion has no effect on epithelial homeostasis. ΔNp63 also drives adult luminal cells toward a basal-like fate through an intermediate hybrid multipotent state, reinforcing the concept of stem cell plasticity^48^. Together, TA-p63 and ΔN-p63 regulate the balance between self-renewal and differentiation that maintain normal mammary gland homeostasis, and disruption of this balance can promote TIC formation. TA-p63 exerts tumor-suppressive activity primarily by inducing senescence and apoptosis and by reducing metastasis^49^. By contrast, ΔN-p63, the predominant isoform in basal-like and triple-negative breast cancers, enhances tumor initiation through multiple pathways, including FZD7-mediated Wnt activation^46^, and direct activation of Hedgehog pathway components such as conic hedgehog, GLI2, and Patched1 (PTCH1)^50^.

## EMT-associated transcription factors

Epithelial–mesenchymal transition (EMT)-associated transcription factors such as Slug (SNAI2), Snail (SNAI1), TWIST1/2, and SOX family members, are key regulators of mammary epithelial plasticity and are frequently reactivated in breast cancer, where they promote tumorigenesis, invasion, and metastasis^51^. Co-expression of Slug and Sox9 is sufficient to convert differentiated luminal cells into MaSC-like cells, and this same transcriptional duet drives the acquisition of tumor-initiating properties in breast cancer^52^. Snail and Twist similarly induce dedifferentiation, suppress luminal differentiation programs, and enhance cellular motility and stress survival, thereby expanding stem-like pools^53,54^. An SOX2–SOX9 regulatory axis further reinforces stemness, with SOX9 acting downstream of SOX2 to sustain the ALDH⁺ MaSC population and support TIC maintenance and lineage plasticity^55^. SOX10 has also been identified as a key regulator linking stem/progenitor activity to epithelial plasticity and mesenchymal features during mammary development and tumorigenesis^56^.

## miRNA

MicroRNAs (miRNAs) are critical intrinsic regulators of both MaSC activity and TIC function. By post-transcriptionally controlling target mRNAs, miRNAs fine-tune stemness, differentiation, and cellular plasticity in a cell-autonomous manner. miR-199a enhances stemness in both MaSCs and TICs by inhibiting the nuclear receptor co-repressor LCOR, thereby attenuating interferon-induced differentiation and senescence. Furthermore, miR199a–LCOR axis is activated in breast tumors, particularly in ER-negative subtypes, where it promotes tumorigenesis and metastasis^57^. By contrast, the miR-200 family, particularly miR-200c, acts as a potent negative regulator of stemness by targeting EMT-associated transcription factors such as BMI1, ZEB1, and ZEB2. Accordingly, miR-200 expression is reduced in human breast tumors relative to normal mammary tissue^58^. miR-200 has a context-dependent role in metastasis that reflects the dynamic nature of EMT and mesenchymal–epithelial transition. During early metastatic dissemination, downregulation of miR-200 facilitates EMT, local invasion, and intravasation. However, at distant sites, re-expression of miR-200 promotes epithelial re-differentiation and colonization by enabling mesenchymal–epithelial transition and supporting proliferative outgrowth of metastatic lesions^59^.

## Elf5

E74-like factor 5 (ELF5), a member of the E-twenty-six transcription factor family, is essential for luminal lineage specification^60^. Mammary gland-specific Elf5 knockout mice display a complete failure of lactogenic differentiation and alveolar morphogenesis during pregnancy. Loss of Elf5 leads to expansion of the MaSC compartment in both virgin and pregnant glands, largely through increased SLUG expression, activation of Notch signaling, and suppression of STAT5 activity. Similarly, in breast cancer, ELF5 drives the differentiation of TICs, leading to reduced self-renewal capacity and diminished tumorigenic and metastatic potential^61^.

## Major signaling pathways

Wnt signaling is crucial for mammary gland morphogenesis, stem cell maintenance, and breast cancer progression. WNT ligands, including WNT3A, WNT4, WNT5, WNT6, and WNT7, serve as initiating signals of this cascade^62^. Wnt4 is induced by progesterone in luminal cells, and Wnt4^−/−^ mice exhibit ductal branching defects similar to those observed in progesterone receptor knockout mice, underscoring its essential role in ductal branching during early to mid-pregnancy. WNT1 can partially compensate for the branching defects caused by PR loss^63^. Conditional Wnt4 ablation further demonstrates that WNT4 is required for perinatal and pubertal ductal expansion, whereas WNT5A, acting through the non-canonical receptor ROR2, inhibits branching outgrowth in the mammary gland^64^. Canonical Wnt signaling is mediated by Frizzled (FZD) receptors and the co-receptors, lipoprotein receptor-related proteins (LRP) 5/6. FZD7, a direct transcriptional target of ΔNp63, enhances Wnt activity and promotes MaSC function^46^. R-spondins (RSPOs) potentiate Wnt signaling through LRP phosphorylation, with RSPO1 cooperating with WNT4 to enhance MaSC potential^65^. By contrast, AXIN2 acts as a negative feedback regulator, and Axin2-deficient MaSCs exhibit heightened Wnt responsiveness and improved self-renewal capacity^62^. In breast cancer, WNT ligands such as WNT1, WNT3A, and WNT10B are frequently upregulated, driving β-catenin nuclear accumulation and activation of stemness-associated genes, including Myc, Ccdn1, and Axin2 (ref. ^66^). As in MaSCs, ΔN-p63/FZD7 and RSPO/LGR5 signaling enhances TIC activity^46^. The stem cell marker PROCR is also expressed in breast cancer, and its function depends on Neuropilin-1, whose loss suppresses MMTV-Wnt1-driven tumorigenesis^67^. Conversely, Wnt antagonists such as Dickkopf-1/3 (DKK1/3) constrain TIC self-renewal, and Dkk1 is specifically regulated by miR-3 (ref. ^9^).

Besides Wnt signaling, several conserved developmental pathways collectively regulate MaSC function and are frequently subverted in TICs, including Notch, nuclear factor (NF)-κB, Hedgehog, Hippo, and JAK-STAT (Fig. 2a). Notch signaling (NOTCH1–4 and Jagged/Delta-like ligands) controls MaSC fate by promoting luminal lineage commitment and maintaining progenitor identity through downstream effectors such as HES1 and HEY1 (ref. ^68^). NF-κB activity, driven by progesterone-induced RANKL, supports MaSC survival and self-renewal, whereas hyperactive NF-κB in TICs drives inflammatory signaling, EMT, and therapy resistance^69^. Hedgehog signaling, mediated by the Patched receptor (PTCH1), Smoothened (SMO), and GLI transcription factors (Gli1/2/3), has a limited role in adult MaSCs but becomes aberrantly activated in TICs, where it enhances proliferation and tumorigenicity^70^. The Hippo pathway, through MST–LAST–YAP/TAZ axis, regulates MaSC proliferation, self-renewal, and ductal morphogenesis, whereas dysregulated YAP/TAZ activity in breast cancer promotes TIC self-renewal, EMT, metastasis, and chemoresistance^71^. The JAK–STAT pathway, particularly STAT3 and STAT5, integrates cytokine and hormonal cues to regulate MaSC expansion, alveologenesis, and lineage specification. Constitutive activation of STAT3 in breast cancer sustains TIC maintenance, immune evasion, and metastatic potential^72^. Together, these pathways form an interconnected regulatory network that maintains MaSC function but, when dysregulated, fuels TIC emergence and persistence.

## Microenvironmental components

The mammary microenvironment, or niche, comprises adipocytes, vasculature, ECM, and diverse stromal and immune cell types that collectively shape the behavior, plasticity, and fate of both MaSCs and TICs (Fig. 2a).

## Fibroblast

Throughout mammary gland development, stromal fibroblasts have a crucial role in directing ductal morphogenesis and coordinating ECM remodeling. During epithelial elongation and branching, fibroblasts facilitate MaSC function by secreting various growth factors, including FGF, IGF, HGF, PDGF, and TGF-β1 (refs. ^1,51^). Among these factors, FGF2 and FGF9 activate ERK1/2 signaling in MECs to promote cell proliferation^73^. Fibroblasts also potentiate Hippo and Hedgehog pathway activity through mechanisms including GLI2 expression and increased YAP or TAZ to modulate MaSC and TIC behavior^70,71^. Crucially, the physical expansion of the mammary tree is contingent upon the proteolytic remodeling of the dense stroma, and loss of key matrix metalloproteinases, such as MMP2 or MMP3, results in significant defects in ductal branching and the penetration of the mammary fat pad^5,74^. In addition, fibroblast-derived epimorphin, released in a metalloproteinase-dependent manner, induces epithelial MMP2, MMP3, and C/EBPβ expression to support branching morphogenesis^5^. The Sprouty (Spry) gene family, particularly Spry1 and Spry2, functions as negative feedback regulators of receptor tyrosine kinase signaling. Loss of Spry genes results in cell-autonomous hyperactivation of EGFR-ERK and FGFR pathways in stromal fibroblasts, thereby accelerating epithelial branching^73^. Consistently, reduced SPRY expression across major human breast cancer subtypes underscores its tumor-suppressive role and is associated with enhanced TIC properties.

## Macrophage

Mammary tissue-resident macrophages actively shape epithelial stem cell behavior by integrating developmental, hormonal, and inflammatory cues into signals that regulate MaSC maintenance and TIC expansion^4,6^. Early evidence for the essential role of macrophages in mammary gland development came from studies of colony-stimulatory factor 1 (CSF1) signaling. CSF1-deficient (Csf1^op/op^) mice exhibit a marked depletion of macrophages and display impaired ductal elongation and branching morphogenesis, establishing macrophages as critical regulators of mammary development^1,51^. Building on this foundational evidence, more recent mechanistic studies have further defined macrophage–epithelial crosstalk. The Notch ligand Delta-like 1 (DLL1) is enriched in MaSCs, where it engages NOTCH receptors on neighboring mammary macrophages. This DLL1–NOTCH signaling activates the Notch and Wnt coupling pathways within macrophages, thereby generating a positive feedback loop that supports MaSC activity^24^. A distinct population of niche-resident CXCR4⁺ macrophages has also been identified. These cells respond to luminal-derived CXCL12 and produce MMP2, WNT2B, and Cyclin D1, thereby enhancing the regenerative capacity and survival of both MaSC and TICs^75^. In addition, ex vivo organoid studies show that macrophage-derived tumor necrosis factor-α promotes MaSC function through PI3K/CDK1/Cyclin B1 signaling cascade^76^. Under cytotoxic stress, ductal macrophages interact with PROCR⁺ MaSCs via IL-1β/IL-1R/NF-κB signaling to maintain stem cell survival and function^77^. In the tumor microenvironment, TICs directly engage tumor-associated macrophages through CD90–CD11b, LSECtin–BTN3A3, and EPHA4–ephrin interactions, establishing an immunosuppressive niche that drives tumorigenesis^78^.

## T cell

The adaptive immune system also contributes to mammary epithelial differentiation. CD4^+^ T cells act as negative regulators of mammary gland morphogenesis. Specifically, CD11c^+^ antigen-presenting cells activate CD4^+^ T helper 1 (T_H_1) cells, leading to interferon-γ secretion that modulates luminal lineage. By contrast, T_H_2 cells exert opposing effects on ductal development, underscoring the importance of T cell balance in maintaining epithelial hemostasis differentiation^79^. Foxp3^+^ regulatory T cells have been shown to confer immune privilege to breast cancer TICs, although their role in normal mammary epithelial regulation remains less clear^75^. In addition, exhausted CD8⁺ T cells can impair the efficacy of PD-1 blockade and support the maintenance of TICs^80^.

## Adipocyte

Adipocytes are the most abundant stromal cell type in the breast^1^. Although their primary role in the healthy mammary gland involves structural support and providing lipid during lactation, they become a central hub for metabolic and inflammatory signaling in the context of breast cancer. By secreting a diverse array of adipokines, lipids, and inflammatory mediators, such as leptin, IL-6, and tumor necrosis factor-α, adipocytes promote EMT-like programs, enhance stem-like properties, and support tumor-initiating capacity^81^. Adipocyte-derived fatty acids are directly sequestered by tumor cells to fuel metabolic reprogramming, sustaining proliferation and survival even under nutrient-depleted stress conditions. Cancer-associated adipocytes exhibit a dedifferentiated, inflammatory phenotype, in concert with stromal and immune cells, further sculpting a pro-tumorigenic niche that reinforces chronic inflammation and immune modulation^81^.

Together, these intrinsic, signaling, and microenvironmental regulators highlight how mammary stemness is governed by a highly dynamic and context-dependent network. Because many of these regulatory circuits are sensitive to physiological remodeling, life stages such as pregnancy and aging can transiently reprogram the mammary niche and alter susceptibility to malignant transformation. The following section explores how these physiological windows create unique vulnerabilities that influence breast cancer risk.

## Pregnancy and postpartum involution: mammary remodeling and windows of tumor susceptibility

Breast cancer is the most common malignancy diagnosed in association with pregnancy in the USA. Although parity confers a long-term reduction in lifetime breast cancer risk, there is a transient increase in risk for women immediately after childbirth. Across age groups, parous women exhibit a higher incidence of breast cancer compared with nulliparous women^82,83^. This is known as the “dual” effect of pregnancy, in which pregnancy causes a period of promotion followed by its long-term protective effect. In fact, having a completed pregnancy within 5 years before diagnosis is considered an independent, negative prognostic factor, even after controlling for tumor size and stage^84^. Breast cancer diagnosed during pregnancy, lactation, or within 1 year after childbirth is classified as pregnancy-associated breast cancer (PABC)^85^. Epidemiologic studies show that patients with PABC have worse clinical outcomes and more aggressive tumor features, including larger tumor size, higher grade, and more advanced stages, than non-PABC patients, with the poorest prognosis occurring during the postpartum period when PABC incidence peaks^86^. In recent years, breast cancer diagnosed specifically in the postpartum period has gained recognition as a distinct clinical entity within the broader PABC spectrum. Postpartum breast cancer (PPBC), typically defined as breast cancer diagnosed within 10 years after childbirth, is associated with more aggressive disease and significantly higher rates of metastasis and mortality^82^. This epidemiologic pattern has shifted attention toward postpartum physiological processes that may create a transient window of heightened tumor susceptibility.

A defining feature of the postpartum period is mammary gland involution during which the gland undergoes extensive epithelial cell death and stromal restructuring to return to its pre-pregnancy state^2,3^. In both rodents and humans, involution resembles a wound-healing program marked by immune cell influx^87^, ECM remodeling^88^, and lymphangiogenesis^89^. These processes are known to involve tumor growth and progression, forming the basis of the involution hypothesis that the postpartum involution microenvironment is tumor promotional. Experimental models demonstrate that the involuting gland accelerates tumor growth and metastasis through ECM remodeling and the release of growth factors that enhance tumor proliferation and invasion^88^. The increased density of lymphatic vessels and vasculature, driven by involution-associated lymphangiogenesis, further creates conditions favorable for cancer cell dissemination and lymph node metastasis^89^. Normal postpartum involution is associated with marked increases in immune infiltration, including neutrophils, macrophages, eosinophils, T cells, B cells, and plasma cells^87^. Patients with PPBC similarly exhibit elevated levels of infiltrating CD4^+^ and CD8^+^ T cells, T follicular helper cells, B cells, and natural killer cells compared with nulliparous women^90^. However, these infiltrates are highly skewed toward immune tolerance rather than effective antitumor activity. PPBC tumors show an accumulation of FoxP3^+^ regulatory T cells and upregulation of T cell exhaustion markers such as PD-1 (ref. ^91^), reflecting a profoundly immunosuppressed tumor microenvironment. Macrophages recruited to the involuting gland display tumor-associated macrophage-like features and promote tumor cell motility, invasion, and lymphangiogenesis, contributing to high metastatic propensity of PPBC^92^. Moreover, patients with low immunoglobulin gene signatures but high plasma B cell infiltration have an increased risk of metastasis and mortality, suggesting a potential pro-tumor role for specific B cell states in the PPBC setting^93^ (Fig. 2b).

Collectively, these studies identify postpartum involution as a critical biological window during which coordinated immune and stromal remodeling creates a microenvironment highly permissive to tumor progression. Although relatively less studied, PPBC involves distinct epithelial gene programs, including activation of the STAT1–GBP1 axis, COX-2, and SEMA7, which promote proliferation and a pro-inflammatory microenvironment, increasing susceptibility to malignant transformation^87,94^. Conversely, post-pregnancy induces cell-autonomous epigenetic reprogramming of MECs, rendering post-pregnancy MECs resistant to cMyc-driven oncogenic programs and carcinoma initiation^95^, indicating a protective effect of parity. Together, these pregnancy-associated epithelial changes underscore the need for further investigation. In addition, the specific cellular subsets and molecular pathways that drive PPBC remain incompletely defined, necessitating higher-resolution mapping of the postpartum immune and stromal landscape.

## Aging-associated remodeling of the mammary gland and tumor susceptibility

Aging profoundly alters the cellular and molecular landscape of the mammary gland, leading to structural, transcriptional, and functional changes that predispose to tumorigenesis. During aging, the TDLUs of the breast undergo gradual regression, whereas the proportion of adipose tissues continues to increase. Several studies have similarly shown that postmenopausal breast adipose tissue expands more rapidly with age, accompanied by a reduction in parenchymal components^96^. These structural alternations may contribute to hormonal fluctuations and an abnormal increase in pro-inflammatory factor secretion, potentially leading to a higher incidence of breast cancer.

The mammary gland is composed of diverse cell types, and age-associated changes have been observed in both epithelial and stromal components. Recent single-cell transcriptomic analysis of aged mouse mammary tissue reveals that aging reprograms epithelial, stromal, and immune cell populations^97^. Among epithelial cells, alveolar luminal cells markedly expanded with age, whereas hormone-sensing luminal cells declined. Although basal–myoepithelial cells showed only a modest numerical increase, they underwent striking transcriptional reprogramming, characterized by upregulation of cytokine and growth factor genes and downregulation of genes related to metabolism, ECM organization, and cytoskeletal contractility. In the stromal compartment, aging was associated with a decrease in fibroblast proportion and reduced expression of ECM genes, consistent with previous findings in the human breast, a loss of connective tissues. Furthermore, the proportion of lymphoid cells, including CD8^+^ T and natural killer cells, declined with age, whereas myeloid cells with tumor-associated macrophage-like features expanded^97^. A study exploring age-related changes in the rat mammary gland further confirmed substantial alternations in epithelial and lymphocyte populations, revealing aberrant epithelial proliferation and a reduction in naive B and T cells, reminiscent of precancerous changes^98^. Mechanistically, this study identified midkine (MDK), a heparin-binding growth factor secreted by basal epithelial cells, as a mediator of age-associated molecular shifts that promote MEC proliferation. Importantly, higher MDK levels were observed in ductal carcinoma in situ and IBC patient samples compared with normal breast tissue, and younger patients with breast cancer with high MDK-expressing ER+ tumors exhibited significantly shorter disease-specific survival, indicating that MDK serves as an age-dependent predictor of breast cancer risk. Overall, these findings, together with recent studies in various murine models^99^ and human breast tissues^100^, underscore the dynamic cellular heterogeneity and distinct microenvironmental remodeling that distinguish young from aged mammary glands. In particular, the loss of strict luminal lineage identity, coupled with stromal senescence, ECM remodeling, and elevated systemic and local expression of pro-inflammatory and growth factors such as MDK, emerges as a major driver linking aging with increased breast cancer susceptibility (Fig. 2c).

## Conclusion

Here, we summarize current knowledge and recent advances that have reshaped our understanding of MEC types, their potential lineage relationships, and the intrinsic and microenvironmental regulators that govern their physiological functions and susceptibility to malignant transformation. Large-scale single-cell and spatial profiling, together with functional studies, has revealed a far greater epithelial diversity than previously recognized, including dynamic transcriptional programs and transient intermediate populations that challenge the classical, rigid hierarchical model. Life history-associated changes, including pregnancy, aging, metabolic status, and environmental exposures, further remodel the mammary epithelial landscape and its stromal and immune niches. These physiological transitions create windows of heightened plasticity, vulnerability, or resilience that can either increase breast cancer risk (for example, postpartum involution, aging, and obesity) or confer long-term protection (for example, early full-term pregnancy). Ongoing efforts aimed at defining the spatiotemporal dynamics of MaSCs, progenitors, and their niche signals will help clarify the roots of breast cancer heterogeneity and inform strategies to overcome therapeutic resistance and metastasis.
